# Non-Interventional Factors Influencing Velopharyngeal Function For Speech In Initial Cleft Palate Repair: A Systematic Review Protocol

**DOI:** 10.1186/s13643-019-1141-2

**Published:** 2019-11-05

**Authors:** David Sainsbury, Caroline Williams, Catherine de Blacam, Joanne Mullen, Ambika Chadha, Yvonne Wren, Peter Hodgkinson, Cleft Multidisciplinary Collaborative, Bronia Arnott, Suzanne McDonald, Fiona Beyer

**Affiliations:** 1 Newcastle upon Tyne Hospitals NHS Foundation Trust, Newcastle upon Tyne, UK; 2 Royal College of Surgeons in Ireland, Dublin, Ireland; 3 Guys’ and St. Thomas’ NHS Foundation Trust, London, UK; 4 Bristol Dental School, University of Bristol, Bristol, UK; 5 Bristol Speech and Language Therapy Research Unit, North Bristol NHS Trust, Bristol, UK

**Keywords:** Cleft palate, Palatoplasty, Speech, Velopharyngeal insufficiency, Hypernasality, Velopharyngeal incompetence, Velopharyngeal dysfunction, Poor speech, Fistula, Speech surgery

## Abstract

**Background:**

This systematic review aims to inform the development of a screening tool which pre-operatively predicts which children are likely to develop velopharyngeal insufficiency, one of the causes of poor speech outcomes, following cleft palate repair. This would be highly beneficial as it would inform pre-operative counselling of parents, allow targeted speech and language therapy, and enable meaningful comparison of outcomes between surgeons, techniques, and institutions. Currently, it is unclear which factors influence speech outcomes. A systematic review investigating the non-interventional factors which potentially influence speech outcomes following cleft palate repair is warranted. This may be illuminating in itself or provide foundations for future studies.

**Methods:**

A systematic review will be carried out according to Cochrane methodology and reported according to PRISMA guidelines (PLoS Med 6: e1000097, 2009). Systematic review software will be used to facilitate three-stage screening by two independent reviewers experienced in cleft lip and palate. Thereafter, data extraction and GRADE assessment will be performed in duplicate by five independent reviewers experienced in cleft lip and palate. Studies reporting the proportion of patients who were recommended or underwent secondary speech surgery for velopharyngeal insufficiency following primary surgery for cleft palate will be included.

The study findings will be tabulated and summarised. The primary outcome measure will be further speech surgery (either recommended or performed). The secondary outcome measure will be perceptual speech assessment for the presence of velopharyngeal insufficiency. A meta-analysis is planned. However, if this is not possible, due to the anticipated marked heterogeneity of study characteristics, pre-operative assessment, and the recorded outcome measures, a narrative synthesis will be undertaken.

**Discussion:**

This systematic review may provide sufficient data to inform the development of a screening tool to predict the risk of velopharyngeal insufficiency prior to cleft palate repair. However, it is anticipated that these findings will provide the foundation for future studies in this area.

**Systematic review registration:**

Registered on 19 December 2016 with PROSPERO CRD42017051624

## Background

Cleft palate is one of the commonest congenital craniofacial conditions, affecting 1 in 2000 live births [2]. The aims of cleft palate repair are the anatomical reconstruction of an intact palate and a functional velopharyngeal mechanism to allow for normal speech development and feeding. In addition to poor speech, other complications following cleft palate repair include fistula, bleeding, and iatrogenic impact on mid-facial growth [12]. Consequently, outcomes following cleft palate repair may be assessed in terms of palatal fistula (an abnormal communication between the oral and nasal layers of the palate), the incidence of velopharyngeal insufficiency (the inability to make a functional seal between the nose and mouth resulting in characteristic, hypernasal cleft type speech with air audibly leaking down the nose), cleft speech characteristics (which measures articulation) and facial growth analysis.

Reported rates of velopharyngeal insufficiency range from 0 to 66% [17]. Possible factors responsible for this disparity include the broad clinical phenotype of orofacial clefting, the range of classification systems used in assessing cleft severity, the inclusion of multiple surgeons, techniques, or institutions within the same studies, and the inherent limitations of retrospective data collection in many studies [8].

It would be highly beneficial if it were possible to pre-operatively determine which children were likely to develop velopharyngeal insufficiency (leading to poor speech outcomes) following their cleft palate repair. Firstly, this would facilitate pre-operative counselling of parents before a palate repair. The families are informed pre-operatively about the risk factors and likelihood of success or the potential need for secondary surgery or further intervention. Secondly, it would allow extra, targeted care to be given to these children in the form of speech and language therapy. Thirdly, it would enable meaningful, standardised comparison of cleft repair techniques, surgeons, and cleft centres undertaking palatal surgery to improve national and international standards.

It is likely that a diverse range of factors influence the success of a cleft palate repair with respect to speech and other outcomes. Male sex, greater cleft width, and shorter palate length have been shown to be independent predictors of velopharyngeal insufficiency [8, 11, 13]. Combined cleft lip and palate was associated with wider mean cleft palate width and a higher incidence of shorter palates than in isolated cleft palate [8]. Velopharyngeal insufficiency was more frequent in cases of combined cleft lip and palate than in cleft palate alone [8]. However, this was a single institution and single surgeon study and, other than gender and presence of lip involvement, was based on factors which could only be accurately established at the time of surgery (i.e. were based on the anatomy of the cleft palate). A systematic review has not been undertaken looking at the non-interventional factors or exposures which influence outcomes following cleft palate repair. Interventions include any surgery, any medications including antibiotics, non-surgical devices such as naso-alveolar moulding or arm splints, speech, and language therapy input.

### Aim

The question this systematic review is aiming to answer is what non-interventional factors influence poor velopharyngeal function for speech (as indicated by the proportion of patients being recommended or undergoing re-operation or speech surgery) following initial cleft palate repair?

## Methods/design

A systematic review will be carried out according to Cochrane methodology and reported according to PRISMA guidelines [9]. This was registered on 19 December 2016 with PROSPERO CRD42017051624. Systematic review software (Distiller SR, Evidence Partners, Ottawa, ON) will be used to facilitate three-stage screening by two independent reviewers experienced in cleft lip and palate. Data extraction will be performed in duplicate by independent reviewers experienced in cleft lip and palate. Studies that report the proportion of patients who were recommended or underwent secondary speech surgery for velopharyngeal insufficiency following primary surgery for cleft palate will be included. The study findings will be tabulated and summarised. The primary outcome measure will be further speech surgery (either recommended or underwent). The secondary outcome measure will be perceptual speech assessment for the presence of velopharyngeal insufficiency. Any protocol deviations which occur during the review will be reported and the reasons behind it in the section “Differences between Protocol and Review” of the systematic review.

### Eligibility criteria

The objectives for this systematic review are defined using a PECO framework (Table 1) [10].

**Table 1.** PECO table

Criteria	Description
Patients/population	Any patient who has undergone cleft palate repair. Inclusion criteria: 1. Studies with 10 or more participants 2. Human studies 3. Patients undergoing initial cleft palate repair 4. Patients with or without a syndrome; with or without a genetic anomaly Exclusion criteria: 1. Studies containing less than 90% participants with cleft palate (with or without cleft lip) 2. Non-human studies 3. Studies with patients with isolated cleft lip only
Exposures	Any exposure (or non-interventional factors) which may influence speech outcomes including gender, cleft phenotype, timing of cleft palate surgery, presence of syndrome, or Robin sequence (please see text below)
Comparison	Exposures* will be compared within each sub-category, i.e. for gender, timing of surgery, cleft phenotype, presence of syndrome, or Robin sequence
Outcomes	1. The proportion of patients who had or were recommended further surgery for velopharyngeal insufficiency (i.e. proportion of patients undergoing or who were recommended re-operation or speech surgery) 2. The proportion of patients with normal velopharyngeal function for speech following initial cleft palate repair

*For more information please refer to the “Comparator(s)/Control” section.

### Participants/population

There will be no exclusion based on the year of publication, and databases will be searched from inception (Table 1).

### Exposure(s)

Any surgical intervention and schedule will be included in the review.

Of interest are the following:

1. Non-cleft related pre-operative factors
  - Gender
  - Ethnicity
  - Premature birth
  - Age at time of soft palate repair
  - Age at time of hard palate repair
  - Syndromal presence/genetic anomaly
  - Birth weight
  - Weight at palate repair
  - Head circumference
  - Robin sequence
  - Deprivation index
  - Socio-economic status
  - Hearing problems
  - Developmental delay
  - Previous general anaesthetics
  - Significant associated comorbidities (e.g. cardiac, respiratory and airway issues, feeding issues)
2. Cleft-related pre-operative factors
  - Cleft type
    i. Bilateral cleft lip-cleft palate
    ii. Unilateral cleft lip-cleft palate
    iii. Cleft palate only
    iv. Cleft of the soft palate
    v. Sub-mucous cleft palate
  - Unilateral or bilateral
  - Lip involvement
  - Alveolar involvement
  - Hard palate involvement

### Comparator(s)/control

Exposures will be compared within each sub-category, i.e. gender, timing of surgery, cleft phenotype, presence of syndrome, or Robin sequence. In this systematic review, we are interested in non-interventional factors or exposures which influence speech outcomes following cleft palate repair. For example, if the exposure is one gender, then the comparator is the other gender; if the exposure is timing of surgery less than *N* months, the comparator is timing of surgery more than *N* months; if the exposure is the presence of a syndrome, comparator is no syndrome, etc.

### Context

Reported rates of velopharyngeal insufficiency vary widely following initial cleft palate surgery. For example, published rates of VPI range from 0 to 66% (Smith and Losee 2014). Whilst certain factors have been implicated (e.g. male sex, bilateral cleft lip and palate, cleft width), the reason for this variation is unclear. The factors of interest have been included as there is some low quality or equivocal evidence that they may be associated with poor speech outcomes. For example, socio-economic deprivation has been suggested by some to be associated with poor speech outcomes [16] but not by others [4]. Consequently, the reason for including many of the factors of interest is that there is some evidence they impact speech outcomes following cleft palate repair, such as cleft type or age at surgery, or realistically could do so, such as Robin sequence or previous general anaesthesia) [15, 18].

### Outcome(s)

The principal interest of this review is the need for further surgery with the aim to improve speech following initial cleft palate repair.

Whilst theoretically the incidence of velopharyngeal insufficiency might be considered the best primary outcome measure there is wide variability in type, use, and interpretation of perceptual speech assessment tools. Consequently, there is heterogeneity across the literature with respect to recorded speech parameters. It is therefore unlikely that it will be realistically possible to pool or compare these. Therefore, the rate of secondary speech surgery (including cleft palate re-repair, pharyngoplasty, pharyngeal wall augmentation, or palate lengthening procedures such as buccinator myomucosal flaps) will be used as an indicator for poor speech outcomes. This must be interpreted with a degree of caution as there will be some children with poor speech outcomes who do not proceed to further speech surgery. Additionally, the threshold for secondary speech surgery is likely to vary between centres, clinicians, families, and patients.

### Primary outcomes

The primary outcome will be the proportion of patients who had or were recommended further surgery for velopharyngeal insufficiency (i.e. proportion of patients undergoing or who were recommended re-operation or speech surgery). This includes patients undergoing cleft palate re-repair (include palate muscle re-positioning, secondary intravelar veloplasty, and Furlow’s double-opposing z-plasty), pharyngoplasty (pharyngeal flaps, sphincter pharyngoplasty), pharyngeal wall augmentation (with cartilage, fat or silicone), or palate lengthening procedures such as buccinator myomucosal flaps.

### Secondary outcomes

The secondary outcome measure is the proportion of patients with normal velopharyngeal function for speech following initial cleft palate repair. This includes measures such as resonance and nasal emission. It is anticipated that it may be challenging to pool such variables as the recorded speech parameters have wide heterogeneity in terms of recording the perceptual speech assessment. There will also be a variation between cleft teams and clinicians as to the definition of “normal” speech.

Effect measures will be recorded using perceptual assessment scales. The definition of improvement will depend on which perceptual assessment scale each study has used. VPI assessment is assessor-dependent, using tools such as the Great Ormond Street Speech Assessment Tool [14]. Objective tools to assess VPI such as nasometry are generally only used for research purposes. Any speech assessment will be included in the review to capture all relevant papers. However, studies will be excluded if the reported speech outcomes do not relate to velopharyngeal insufficiency. Studies reporting only psychosocial outcomes will be excluded.

To facilitate comparison between studies, we will record resonance outcomes for each study under five categories—normal, mild hypernasality, moderate hypernasality, severe hypernasality, or hyponasality. If it is not possible to determine the degree of hypernasality, it will be recorded as “hypernasality of unknown severity”. Nasal airflow errors (emission and turbulence) will be recorded as normal, mild, moderate, or severe. If the authors have not stratified this then this will be recorded as ‘presence of nasal emission’, ‘presence of nasal turbulence’, ‘no airflow errors’, or ‘not reported’.

### Follow-up length

To be included in the review, studies must report the proportion of patients who were recommended or underwent secondary speech surgery for velopharyngeal insufficiency at five years of age or older. This length of time is necessary to allow time for the child’s speech and language skills to develop as part of a typical trajectory in English speaking children [5].

### Information sources

A comprehensive electronic database search from inception until present date will be performed using the following:

1. MEDLINE (OVID interface, 1948 onwards)
2. EMBASE (OVID interface, 1980 onwards)
3. AMED
4. PsycINFO
5. CINAHL—Cumulative Index to Nursing and Allied Health Literature
6. Health Technology Assessment Database
7. SpeechBITE
8. Cochrane Central Register of Controlled Trials (CENTRAL)
9. Cochrane Database of Systematic Reviews
10. Scopus

If any systematic reviews are retrieved from the Health Technology Assessment Database and Cochrane Database of Systematic Reviews searches, then the reference lists in these articles will be searched for any relevant articles. The systematic review article itself will not be included within this systematic review.

### Search strategy

Literature search strategies will be developed using medical subject headings (MeSH) and text words related to cleft palate. The literature search will include articles written in all languages but will be limited to human subjects. To ensure literature saturation, we will scan the reference lists of included studies or relevant review articles identified through the initial search.

Publications of potential relevance to our study will be identified by using both exploded MeSH headings and text words. The search strategy has been peer-reviewed by two independent librarians, and the final search will be performed by one of these librarians (JM).

cleft*, OR

palat*

AND

repair*, OR

palatoplast*, OR

reconstruct*, OR

surg*, OR

operat*, OR

reconstruct*, OR

pharyngoplast*, OR

pharyngeal flap, OR

palatal surg*, OR

furlow, OR

honig, OR

hynes, OR

orticochea, OR

sanvenero-roselli, OR

jackson OR,

skoog OR,

(fat ADJ inject*)

OR (cartilage ADJ graft*)

OR (fat ADJ graft*) OR

buccinators ADJ flaps, OR

pharyngeal ADJ wall ADJ augmentation

sommerlad repair* OR

intravelar veloplast*, OR

von langenbeck*,

AND

speech ADJ outcome*, OR

velopharyngeal function*,

velopharyngeal ADJ (insufficiency OR dysfunction OR incompeten*), OR

resonance, OR

hypernasal*, OR

passive ADJ CSCs, OR

nasal ADJ (emission OR turbulence OR regurgitation), OR

airflow ADJ errors, OR

speech, OR

nasalance, OR

cleft ADJ speech, OR

poor ADJ speech

We will not include literature reviews, case reports, case series with less than 10 participants, review articles, commentaries, letters, editorials and dissertation abstracts, and conference proceedings. We consider these forms of publication to deliver insufficient information and to present a higher risk of reporting error, as compared with peer-reviewed articles. A preliminary review has been conducted which suggests that there is an extensive evidence base to draw from within the peer-reviewed literature. Therefore, it was felt unnecessary to extend this to encompass the grey literature.

## Study records

### Selection process and study design

Studies will be screened for inclusion using the above inclusion criteria (Table 1). The following types of study will be included:

1. Studies reporting the proportion of patients who were recommended or underwent re-operation or speech secondary surgery for VPI at 5 years of age or older.
2. Studies reporting the proportion of patients with normal velopharyngeal function for speech following initial cleft palate repair.
3. Randomised controlled trials (RCTs), cluster RCTs, non-randomised controlled clinical trials, longitudinal studies with and without controls, cross-sectional investigations with and without controls, and retrospective studies with prospective data collection with and without controls, case series with 10 or more participants.

The following types of study will be excluded:

1. Literature reviews (previous reviews will be identified to establish their findings and therefore, provide a background to previous research).
2. Case reports, case series with less than 10 participants, review articles, commentaries, letters, editorials, and dissertation abstracts.

We will not prioritise one study type over another because the intervention is not what is of primary interest. Consequently, trials and observational studies are of equal importance. However, the outcomes might look different as those from trials will be looking specifically at changes in response to an intervention while those from observational studies would be looking over time at changes. We will report the outcomes from trials and observational studies separately in order to observe any patterns which might be based on the different study designs.

### Screening

The search results will be uploaded to DistillerSR (Evidence Partners, Ottawa, ON) and de-duplicated. Thereafter, a three-stage screening process will be used. During the first stage, based upon titles, two independent reviewers will exclude articles that are evidently not pertinent to the review. In the second stage, two reviewers will read and review all abstracts yielded by the search against inclusion criteria. The co-investigators will retrieve the full text of all articles that appear to meet the inclusion criteria or in any case where there is uncertainty about eligibility. At the third stage, the full-text papers will be reviewed in duplicate. The reasons for excluding papers will be recorded. Discrepancies in study selection will be dealt with by discussion, with unresolved conflicts solved by third-party arbitration. Uncertainties regarding eligibility for inclusion will be clarified by contacting study authors when necessary. To avoid overlapping patient populations, data on authorship, recruitment years, data source, and geographic location will be compared at the data extraction stage. If a patient population is found to overlap, the article with the most comprehensive data will be included. Review authors will not be blinded to the journal titles or to the study authors or institutions. A pilot of the data extraction form will be performed.

### Data collection process

#### Data extraction and analysis

Five independent reviewers will perform data extraction in duplicate. EndNote X7 (Thompson Reuters, Philadelphia, PA) bibliographic software program will be used to manage citations. Screening and data extraction will be completed using DistillerSR (Evidence Partners, Ottawa, ON) online systematic review software.

The following variables will be recorded from each article:

- *Study characteristics*
  - Author(s)
  - Year of publication
  - Dates of study period
  - Funding source
  - Country of origin
  - Type of study design
- *Population characteristics*
  - Procedure
  - Number of patients
  - Gender of patients
  - Ethnicity
  - Adoption
  - Prior surgery
    - Tonsillectomy
    - Adenoidectomy
    - Previous general anaesthetic
  - Mean birth weight
  - Weight at palate repair
  - Premature birth
  - Feeding history
    - Breast-fed
    - Bottle-fed
    - Supplemental feeding, e.g. nasogastric tube
  - Maternal smoking
  - Consanguinity
  - Family history of palatal anomaly
  - Syndrome diagnosis/genetic anomaly/Robin sequence
  - Significant associated comorbidity
    - Hemi-facial microsomia
    - Neurofibromatosis
    - Moebius syndrome
    - Microtia
  - Airway issues or obstructive sleep apnoea
  - Head circumference
  - Deprivation index
  - Socio-economic status
  - Hearing problems
    - Including otitis media with effusions, grommet insertions, use of a hearing aid
  - Developmental delay
  - Cleft type
    - LAHSHAL or LASHAL code
    - Veau (I, II, III, IV)
    - Bilateral cleft lip-cleft palate
    - Unilateral cleft lip-cleft palate
    - Cleft palate only
    - Complete cleft of secondary palate
    - Incomplete cleft palate with hard palate involvement
    - Complete cleft of soft palate with no hard palate involvement
    - Incomplete cleft of soft palate
    - Sub-mucous cleft palate
    - Unilateral or bilateral
    - Lip involvement
    - Alveolar involvement
    - Hard palate involvement
- *Timing of palate repair*
  - Mean age at soft palate repair
  - Mean age at hard palate repair
- Details of the initial surgical procedure(s)
- Mission surgery
- Surgeon’s experience
- *Method of speech assessment*
  - Speech assessment carried out by a speech and language therapist
  - Use of a validated perceptual speech assessment tool
  - Blinded perceptual speech assessment
  - Speech assessment by more than one therapist
- *Follow-up*
  - Mean length of follow-up until speech assessment
  - Mean length of follow-up at the last speech assessment
- *Speech assessment*
  - Method of speech assessment: speech assessment carried out by a speech and language therapist, use of a validated perceptual speech assessment tool, blinded perceptual speech assessment, and speech assessment by more than one therapist
  - Resonance postoperatively
  - Normal resonance postoperatively
  - Normal nasal emission postoperatively
  - Nasal emission postoperatively
  - Hyponasal postoperatively
- *Post-operative course*
  - Complications
  - Requirement for secondary speech surgery
  - Mean length of time until secondary speech surgery
  - Type of further surgery
    - Posterior pharyngeal wall augmentation (including fat injection, calcium hydroxyapatite injection)
    - Palate re-repair (including Furlow’s palatoplasty, pushback, intravelar veloplasty)
    - Pharyngeal flap
    - Sphincter pharynoplasty
    - Buccinator myomucosal flaps

During data extraction, other variables of interest may be recognised. These will then be added to the dataset and the protocol amended accordingly. The methodology used for the recording of outcomes will also be noted and will include the following variables: speech assessment carried out by a speech and language therapist, use of a validated perceptual speech assessment tool, blinded perceptual speech assessment, speech assessment by more than one therapist.

Percentages of patients achieving normal resonance and nasal emission postoperatively will be extracted as standalone variables in the first instance and will be then included with the improved resonance and nasal emission variables.

### Risk of bias and quality assessment in individual studies

Both bias and quality will be assessed in duplicate. Studies with significant results are more likely to be published than studies without significant results (i.e. publication bias). The ROBINS-E tool will be used for assessing risk of bias in observational studies [1]. The methodologic quality of each study will be appraised using modified criteria based on the Cochrane Collaboration’s tool for assessing risk of bias in therapeutic studies [7]. The following domains will be assessed: selection bias (randomisation of patients to a given treatment), detection bias (blinded assessment of outcomes), outcome assessment (recording of key outcomes—in this case, normalised resonance), and attrition bias (inclusion of all patients who received the treatment in question). Quality will be assessed using the Oxford Centre for Evidence-based Medicine 2011 level of evidence guidelines (OCEBM Levels of Evidence Working Group, 2011). GRADE assessment will be performed in duplicate to assess the certainty of the body of evidence [6].

## Data

### Synthesis

Systematic reviews performed in this area [3] have identified significant diversity of methodology and reporting of results. Consequently, it is anticipated that there will be substantial heterogeneity and that a formal meta-analysis will not be feasible. It is expected that a systematic narrative synthesis will be provided with the information presented in the text and tables to summarise and explain the characteristics and findings of the included studies. The narrative synthesis will explore the relationship and findings both within and between the included studies, in line with the guidance from the Centre for Reviews and Dissemination. For each study, the number of patients who achieved each of the outcomes of interest will be recorded. Individual percentages will be calculated using the number of patients in each study as denominator. We will contact the authors of the trials and studies to resolve any missing data.

## Discussion

Currently, it is unclear which factors influence speech outcomes following cleft palate repair. It would be highly beneficial if it were possible to pre-operatively determine which children were likely to develop VPI (leading to poor speech outcomes) following cleft palate repair. This would inform pre-operative counselling of parents, allow targeted speech and language therapy, and enable meaningful comparison of outcomes between surgeons, techniques, and institutions. This systematic review aims to add to the evidence-base so that teams managing children with a cleft palate can continue to improve outcomes and patient care. This systematic review is unlikely to provide sufficient data to enable risk stratification of the speech outcomes of cleft palate repair based on non-operative factors. However, it is anticipated that this systematic review will provide further knowledge in this area and provide a solid foundation for future studies to inform the development of a pre-operative screening tool.

## Data Availability

The datasets used and/or analysed during the current study are available from the corresponding author on reasonable request.

## Abbreviations

CSCs: Cleft speech characteristics
VPI: Velopharyngeal insufficiency
